# Can passive measurement of physiological distress help better predict suicidal thinking?

**DOI:** 10.1038/s41398-021-01730-y

**Published:** 2021-12-02

**Authors:** Evan M. Kleiman, Kate H. Bentley, Joseph S. Maimone, Hye-In Sarah Lee, Erin N. Kilbury, Rebecca G. Fortgang, Kelly L. Zuromski, Jeff C. Huffman, Matthew K. Nock

**Affiliations:** 1 Department of Psychology, Rutgers, The State University of New Jersey, Piscataway, NJ USA; 2 Department of Psychiatry, Massachusetts General Hospital and Harvard Medical School, Boston, MA USA; 3 Department of Psychiatry, Massachusetts General Hospital, Boston, MA USA; 4 Department of Psychology, Harvard University, Cambridge, MA USA

**Keywords:** Human behaviour, Scientific community

## Abstract

There has been growing interest in using wearable physiological monitors to passively detect the signals of distress (i.e., increases in autonomic arousal measured through increased electrodermal activity [EDA]) that may be imminently associated with suicidal thoughts. Before using these monitors in advanced applications such as creating suicide risk detection algorithms or just-in-time interventions, several preliminary questions must be answered. Specifically, we lack information about whether: (1) EDA concurrently and prospectively predicts suicidal thinking and (2) data on EDA adds to the ability to predict the presence and severity of suicidal thinking over and above self-reports of emotional distress. Participants were suicidal psychiatric inpatients (*n* = 25, 56% female, *M* age = 33.48 years) who completed six daily assessments of negative affect and suicidal thinking duration of their psychiatric inpatient stay and 28 days post-discharge, and wore on their wrist a physiological monitor (Empatica Embrace) that passively detects autonomic activity. We found that physiological data alone both concurrently and prospectively predicted periods of suicidal thinking, but models with physiological data alone had the poorest fit. Adding physiological data to self-report models improved fit when the outcome variable was severity of suicidal thinking, but worsened model fit when the outcome was presence of suicidal thinking. When predicting severity of suicidal thinking, physiological data improved model fit more for models with non-overlapping self-report data (i.e., low arousal negative affect) than for overlapping self-report data (i.e., high arousal negative affect). These findings suggest that physiological data, under certain contexts (e.g., when combined with self-report data), may be useful in better predicting—and ultimately, preventing—acute increases in suicide risk. However, some cautious optimism is warranted since physiological data do not always improve our ability to predict suicidal thinking.

## Introduction

In recent years, there has been increased interest in using wearable devices (e.g., smartwatches) to study psychological constructs of interest in the real world, such as detecting signals of distress that may predict the onset of suicidal thoughts [1–3]. Periods of suicidal thinking can occur rapidly and be highly distressing [4–6], possibly escalating quickly to a level that interferes with the cognitive resources needed to ask for help or use skills learned in therapy. If a wearable monitor could passively detect this distressing state, it would provide opportunities for the deployment of *just-in-time* adaptive interventions [7]. Such interventions would be particularly useful for groups of individuals at elevated risk for suicide [8], such as those who have recently discharged from inpatient psychiatric care for suicide risk (the focus of this study).

There is a long history of laboratory research supporting the promise of passively detecting distress that may characterize or precede periods of suicide risk. The psychological experience of distress is reliably associated with sympathetic autonomic activity. This activity can be indirectly detected by observing the small increases in perspiration that occur during an autonomic event [9, 10]. Because sweat is a good conductor of electricity, changes in skin conductance (also called electrodermal activity; EDA) [11] signal when an individual is distressed. Increases in EDA are associated with laboratory-induced distress in the form of social comparision [10] or watching a disturbing film [12]. Individuals at risk for suicide exhibit increased physiological reactivity to stress (i.e., increased skin conductance) [13] and this reactivity distinguishes those who are suicidal from those who are depressed but not suicidal (for review see Sarchiapone et al. [14]). This aligns with clinical observation and increasing empirical findings that periods of high suicide risk tend to be characterized by the high arousal affective states (e.g., agitation [15]) that are potentially most easily detectable by monitoring autonomic activity.

Although there is optimism about the possibility of using wearable monitors to identify periods of risk for suicidal thoughts among those who are at risk for suicide, there are important questions that must be answered about the predictive ability of autonomic arousal before developing interventions that rely on wearable passive sensing. The goal of this study is to begin answering these questions.

### Is passively detected distress associated with periods of suicidal thinking?

It is currently unknown whether passively detected distress (i.e., increased EDA) measured with a wearable device is associated (either concurrently or prospectively) with periods of suicidal thinking. Importantly, what is found in a controlled laboratory environment—the settings used for the vast majority of such studies to date—may not be as clear in the uncontrolled environment of everyday life. For example, changes in temperature, exercise, being in a hot room, and wearing the device incorrectly can all contribute to noise and may do so to the point of degrading the validity of physiological data [16]. Thus, even though wearable devices are becoming cheaper and easier to use, the technology is still prohibitively expensive to deploy on a large scale based on laboratory research alone. Consequently, it is important to establish whether physiological signals of distress as measured with wearable devices are associated with periods (hours and days) of suicidal thinking.

### Does passively detected distress add to our ability to predict periods of suicidal thinking over and above self-reports of affect?

The “gold standard” for assessing elevations in distress associated with suicide risk involves technology like smartphone-based ecological momentary assessment (EMA) which captures self-reported affective experiences and suicidal thinking in the moment throughout the day [17]. In most initial cases, wearables will not be used as a stand-alone method of detecting risk but will rather be paired with the current “gold standard” of EMA. One key step is to see whether passive detection of distress improves the concurrent and prospective prediction of periods (i.e., hours and days) of suicidal thinking beyond self-report alone. This is important from a scalability perspective because it is still cheaper and easier to deploy monitoring solutions that rely solely on self-report (though certainly, self-report monitoring may require more active engagement for patients/participants). If wearable devices do not add to what self-report can tell us, it may suggest decreased utility of using wearables when self-report is available, and participants are willing/able to respond to these questions.

### The present study

In this study, we were interested in (1) the contemporaneous and prospective associations between physiological assessments of distress (i.e., EDA) and self-reported suicidal thinking as well as (2) the incremental predictive validity of physiological assessment of distress above and beyond self-report, across both the presence/absence of suicidal thinking and the severity of suicidal thinking. Since we consider the aims and analyses in this paper to be exploratory, we have few specific hypotheses. Generally, however, we expected that if passively detected physiological distress adds to our ability to predict self-reported suicidal thinking, it would be most likely to do so in cases where the self-report items do not assess states known to be strongly correlated with these same physiological metrics. For example, increases in EDA are more strongly tied to high arousal, rather than low arousal, affect [18, 19]. When self-report items assess high arousal affect, this may contribute to overlap and therefore redundancy between the self-report ratings and the physiological data. Thus, we expect passively-detected distress to improve the characterization and prediction of suicidal thinking when combined with assessments of low arousal versus high arousal affective states, because we hypothesize that passively-detected distress is less redundant with low-arousal affect than with high-arousal affect.

## Methods

### Participants

Participants were 25 adult inpatients who were hospitalized due to suicidal thoughts or suicidal behavior were recruited from an inpatient psychiatry service from July 2019 until March 2020 at Massachusetts General Hospital as part of a Harvard University IRB-approved registered clinical trial (NCT03950765) testing a novel smartphone intervention. Inclusion criteria were (1) admission due to severe suicidal thinking or a suicide attempt, (2) access to a smartphone, (3) willingness to wear the physiological monitor, and (4) absence of any factor that would preclude capacity to consent (e.g., acute psychosis, drug withdrawal), which was independently confirmed by clinical staff. Our sample size was determined based on power analyses that conservatively assumed 50% compliance (three out of six surveys) over 28 days (i.e., 84 responses per participant). We exceeded this number of assessments (97.08 responses per participant).

### Procedures

#### Recruitment/consent/baseline

Eligible and interested participants provided informed consent and completed baseline measures assessing demographics, history of suicidal thoughts and behaviors, and other trait-level factors. We used only the demographic questionnaire from the baseline session in this study. Participants also were asked to install on their phone a set of apps that allowed us to send surveys and retrieve data from the wearable device.

#### Monitoring period

Throughout their inpatient stay and for 28 days afterward, participants were asked to complete on their smartphone six brief surveys per day. (All participants also received during the inpatient period up to three in-person therapy sessions.) The surveys were hosted on Qualtrics and delivered using the LifeData smartphone app. (We used LifeData to deliver Qualtrics surveys because doing so allowed us to have the benefit of the direct customization over randomization of prompts and the aesthetics of the interface allowed in Qualtrics and the delivery methods (e.g., push notifications) available in LifeData.) The surveys were delivered at random times within pre-specified windows. (Because this was a treatment study, 3 of the 6 daily prompts were randomized to include an opportunity to practice the skills learned in treatment. Before and after each skills practice prompt, participants completed a set of questions assessing a variety of affective states (described below). Because this manuscript is not concerned with the effect of the intervention, we used in these analyses the data from the pre-practice prompts, but not the post-practice prompts. The other three assessments included only the assessment items. Thus, the data we used in this study consisted of the responses to the pre-practice prompts and the assessment prompts.) Participants also were asked to wear on their wrist the Empatica Embrace 2 (www.Empatica.com), a physiological monitoring device that assesses movement (via 3-axis accelerometer), orientation (via a gyroscope), skin temperature, and electrodermal activity. It has been well-validated for its consumer use as an FDA-approved seizure detection device and uses similar technology and sensors as other validated [20–22] research-grade wearables made by the same company. The Embrace syncs to a secure cloud server through the Empatica Mate smartphone app. Participants were asked to wear the device 24 h a day, except for when showering or other times when the device could get submerged in water. We suggested participants charge the device while showering.

### Measures and feature creation

#### Affect

At each prompt, participants were presented with a list of affective state labels and a definition for each state. They were asked to rate each label in regard to the current moment on a 0 (not at all) to 10 (very much) scale. Relevant to this study were five specific negative affect states, categorized into high/low arousal based on the circumplex model of affect [23, 24]. There were three low-arousal negative affect states: (1) fatigued, (2) hopeless, and (3) burdensome and two high arousal negative affect states: (4) agitated and (5) angry.

#### Suicidal thinking

We used a three-item measure of suicidal thinking assessing in the present moment, which has been used in our prior studies [5]. The items assessed the strength of (1) urge to die by suicide, (2) the intention to kill oneself at some point during the next day, and (3) the ability to resist the urge to die by suicide. All items were on a 0 (not at all) to 10 (very strong) scale. In line with prior EMA studies [5], we averaged these items to create a suicidal thinking composite with high internal consistency (alpha = 0.82) (Internal consistency was calculated according to Nezlek’s [25] approach that uses an unconditional three-level model with responses nested within measurement occasion nested within people.). The item assessing ability to resist the urge to die by suicide was reverse-coded.

#### Autonomic events

The Embrace records EDA at 4hz using three stainless steel electrodes mounted on the bottom of the watch case. Once these data are transferred to the server, a proprietary algorithm run on the server classifies autonomic events. Specifically, the algorithm identifies increases in skin conductance level (i.e., tonic EDA) that occur in the absence of increases in temperature and movement (since increases in temperature and movement could be signs that increased EDA is due to being in a hot room or physical activity). The algorithm removes any increase in EDA due to a potential “storm” of rapid EDA changes during sleep [26, 27]. Evaluation of this algorithm in 46 adults found high sensitivity (97%) and a low rate of false positives (0.83/day) (Matteo Migliorini, Ph.D., Empatica s.r.l., Email Communication, April 2021).

### Data preparation

Physiological data were collected continuously, meaning that autonomic events could have been recorded at any time of the day. Self-report data were collected six times daily over a participant-defined 14-h period (most participants chose a window that lasted from 9 am to 11 pm). This means we would likely not have self-report data at or near the time of most autonomic events. To address this, we aggregated our data into two levels, hourly (i.e., within-day) and daily (i.e., between-day). Hourly data provide increased granularity and resolution (we can describe hours instead of days with suicidal thinking); however, only 26% of the autonomic events observed occurred within 1 h of an EMA prompt. Daily data allowed us to use all available autonomic event data, although doing so meant that we lost some ability to determine the temporality of responses. To aggregate the data, we computed for each participant-hour (To maintain consistency with the day-level analyses, we created an average in cases where there was more than one response per hour.) or participant-day an average of the affective states and suicidal thinking ratings and a sum score for number of autonomic events. To adjust for between-person variability in response styles, we participant-mean centered all affective state ratings in the aggregated datasets.

### Analytic strategy

#### Models

Because we were interested in the incremental predictive effects of physiological data over various configurations of self-report data, we tested models that included high or low arousal negative affect, with and without physiological data. We analyzed separate models with high and low arousal negative affect because doing so allowed us to test our specific hypotheses about physiological data improving the predictive ability of low arousal negative affect more than high arousal negative affect. We operationalized our outcome variable, suicidal thinking, in several ways. First, we were interested in both the presence (0, 1) and severity of suicidal thinking. Second, we were interested in both the contemporaneous (i.e., predictors and outcomes at the same timepoint) and prospective associations (i.e., suicidal thinking measured at the next assessment/day). Thus, we had four sets of analyses using combinations of binary vs. continuous data and contemporaneous vs. prospective associations. All models were multi-level models with either a normal distribution (models where severity of ideation was the dependent variable) or binomial distribution (models where presence/absence of ideation was the dependent variable) conducted in the *lme4* [28] R package with fixed slopes and participant-centered predictors.

#### Comparison

To compare models, we used metrics provided by the *performance* [29] package. This includes measures of relative (AIC and BIC) and absolute fit (*R*^2^ and RMSE). In many cases, these metrics are aligned but not perfectly correlated. To reconcile potential differences in model fit ranking across metrics we used the *performance* package’s performance score metric which produced a weighted average of all metrics, suitable for use as an overall summary metric of model performance.

(1) *Akaike information criterion (AIC)* and (2) *Bayesian information criterion (BIC)* balances fit (i.e., minimal prediction error) with parsimony (i.e., having the fewest possible predictors) by penalizing models that have so many predictors (AIC) or data points (BIC) as to risk being overfit to the data. *AIC* and *BIC* are useful to determine which model, among a group of models fit to the same data, best fits the data. A model with a relatively lower *AIC/BIC* value is better fitting than a model with a higher *AIC/BIC* value.

(2) *Bayes factor (BF)* compares a set of models to a comparator. Scores of >1 indicate a model with more support than the comparison model.

(3) *Marginal and conditional* *R*^2^ reflect proportion of variability in the dependent variable accounted for by the independent variables(s). Marginal *R*^2^ considers only the effect of the fixed effects in a multi-level model. Conditional *R*^2^ includes random effects (in this case, the difference in between-person intercepts).

(4) *Root mean squared error (RMSE)* refers to the standard deviation of the error (i.e., deviation between observed and predicted values). Lower values indicate better fit (i.e., less error) and are in the same scale as the dependent variable.

(5) *Performance score* is used to rank the performance of multiple models that takes into account multiple metrics that may not always converge on the same “best” fitting model. It is calculated by rescaling all of the other indices mentioned above from 0 to 1 and then taking the mean across all indices for each model. Thus, scores can range from 0% (all indices point to this model being the worst fitting) to 100% (all indices point to this model being the best fitting).

## Results

The sample consisted of 25 adults (56% female; 44% male. *M* age *=* 33.48 years, *SD* = 13.84 years, 19–63). The sample was 64% White, 20% Asian, 8% Black/African American, and the remaining 8% were of other or multiple ethnicities. 16% were Hispanic or Latinx. The average length of inpatient stay was 7 days (*SD* = 3.16 days). Participants were enrolled in the study on average 1.94 days after admission to the unit (*SD* = 1.35 days). There were 2427 survey responses across 604 days (*M* = 24.16 days, *SD* = 11.74, range = 3–44, compliance rate = 66.98%). 1739 autonomic events were recorded (*M* = 69.56 events per participant, *Mdn* = 62 events, *SD* = 82.20, range = 1 to 297 events), 453 of which (26%) occurred within an hour of an EMA survey prompt (Of those that did not occur within an hour of an EMA prompt, 16% occurred outside of the normal monitoring hours and the remainder occurred in between prompts.).

### Daily-level models

#### Descriptives

Of the 604 days in which we had at least one survey response, at least one non-zero report of suicidal thinking occurred on 396 days (65.56%) and at least one autonomic event occurred on 314 days (51.99%). There were 200 days in which a non-zero report of suicidal thinking and at least one autonomic event co-occurred (i.e., 63.69% of days where there was an autonomic event also contained a non-zero report of suicidal thinking). There were 196 days (i.e., 49.49% of days where an autonomic event was reported) where an autonomic event occurred the day before a report of suicidal thinking.

#### Presence/absence of suicidal thinking

The first section of Table 1 shows contemporaneous (i.e., same day) models and the second of Table 1 section shows prospective (i.e., next day) models using presence/absence of suicidal thinking as the outcome. Both sets of models had the same interpretation. The models including autonomic events consistently performed poorer than the corresponding models that did not include autonomic events. See sections 1 and 2 of Supplementary Table 1 for the individual regression results.

**Table 1:** Model information and model comparison statistics for daily-level data, ordered by performance.

Model	AIC	BIC	Bayes factor	Cond. *R*^2^	Marg. *R*^2^	RMSE	Performance score
1. Presence/Absence of SI, Contemporaneous Analyses							
Low arousal neg. affective states	237.58	259.43	1023.02	0.96	0.02	0.50	99.95%
Low arousal neg. affect + autonomic events	239.40	265.62	46.25	0.96	0.02	0.50	77.45%
High arousal neg. affective states	255.81	273.29	1.00	0.94	0.02	0.54	38.24%
High arousal neg. affect + autonomic events	257.33	279.18	0.05	0.93	0.02	0.54	32.59%
Autonomic events only	264.72	277.83	0.10	0.91	0.00	0.56	1.14%
2. Presence/Absence of SI, Prospective Analyses							
Low arousal neg. affective states	223.43	244.54	9470.50	0.93	0.01	0.53	99.50%
Low arousal neg. affect + autonomic events	225.23	250.56	4493.17	0.93	0.01	0.52	79.97%
High arousal neg. affective states	231.72	248.61	1.00	0.92	0.00	0.54	29.99%
High arousal neg. affect + autonomic events	233.59	254.70	0.09	0.92	0.01	0.54	27.90%
Autonomic events only	234.55	247.22	0.14	0.91	0.00	0.55	0.72%
3. Severity of SI, Contemporaneous Analyses							
Low arousal neg. affect + autonomic events	2473.50	2504.09	564.49	0.82	0.06	1.75	100.00%
Low arousal neg. affective states	2482.48	2508.70	56.28	0.81	0.05	1.78	75.05%
High arousal neg. affect + autonomic	2492.53	2518.75	0.37	0.81	0.05	1.79	69.59 %
High arousal neg. affective states	2494.91	2516.76	1.00	0.80	0.05	1.81	64.24%
Autonomic events only	2598.69	2616.17	0.00	0.76	0.01	1.99	0.00%
4. Severity of SI, Prospective Analyses							
Low arousal neg. affect + autonomic events	2059.58	2089.13	89.90	0.83	0.04	1.59	100.00%
Low arousal neg. affective states	2067.96	2093.29	11.24	0.81	0.03	1.62	68.10%
High arousal neg. affect + autonomic	2074.14	2099.47	0.51	0.82	0.03	1.63	64.58%
High arousal neg. affective states	2077.02	2098.13	1.00	0.81	0.03	1.65	54.33%
Autonomic events only	2126.35	2143.24	0.00	0.79	0.01	1.74	0.00%

Bayes factor reference model was the model with high-arousal negative affect states.

*AIC* Akaike information criterion, *BIC* Bayesian information criterion, *RMSE* root mean squared error.

#### Severity of suicidal thinking

The third section of Table 1 shows contemporaneous (i.e., same day) models and the fourth section of Table 1 shows prospective (i.e., next day) models using severity of suicidal thinking as the outcome. Across all models, adding autonomic events improved model fit and supported our hypothesis regarding greater relative improvements for low versus high arousal negative affect: the improvement in performance score was greater when adding autonomic events to low-arousal negative affect than it was for high arousal negative affect (contemporaneous: 24.95% improvement vs. 5.35% improvement [low vs. high, respectively], prospective: 31.90% vs. 10.25%). See sections 2 and 3 of Supplementary Table 1 for the individual regression results.

### Hourly-level models

#### Descriptives

Of the 2427 survey responses, 1269 (52.29%) had a non-zero score on the suicidal thinking composite. When suicidal thinking was reported, the average severity rating was 5.16 (*SD* = 3.74, max score 30). Of the 453 autonomic events that occurred within an hour of an EMA survey prompt, 160 (35.32%) co-occurred with a non-zero report of suicidal thinking and 172 (37.97%) occurred in the hour before a report of suicidal thinking.

#### Presence/absence of suicidal thinking

The first section of Table 2 section shows contemporaneous (i.e., same hour) models and the second section of Table 2 shows prospective (i.e., next hour) models using presence/absence of suicidal thinking as the outcome. Both sets of models had the same interpretation: autonomic events did not improve the fit of low arousal negative affect. Autonomic events marginally (performance score improved by 0.48%) improved the fit of contemporaneous model with high arousal negative affect. See sections 1 and 2 of Supplementary Table 2 for individual regression results.

**Table 2:** Model information and model comparison statistics for hourly-level data, ordered by performance.

Model	AIC	BIC	Bayes factor	Cond. *R*^2^	Marg. *R*^2^	RMSE	Performance score
1. Presence/Absence of SI, Contemporaneous Analyses							
Low arousal neg. affective states	904.73	933.39	8.66 × 10^14^	0.94	0.02	0.59	99.73%
Low arousal neg. affect + autonomic events	905.20	939.59	3.89 × 10^13^	0.94	0.02	0.59	83.02%
High arousal neg. affect + autonomic events	978.05	1006.71	0.10	0.92	0.01	0.62	31.50%
High arousal neg. affective states	979.25	1002.18	1.00	0.92	0.01	0.62	31.02%
Autonomic events only	1028.19	1045.39	0.00	0.92	0.00	0.64	0.00%
2. Presence/Absence of SI, Prospective Analyses							
Low arousal neg. affective states	255.60	277.20	0.15	0.95	0.00	0.55	73.85%
High arousal neg. affective states	256.12	273.40	1.00	0.95	0.00	0.55	56.78%
Low arousal neg. affect + autonomic events	257.28	283.20	0.01	0.95	0.00	0.55	54.21%
Autonomic events only	256.91	269.87	5.82	0.94	0.00	0.56	40.80%
High arousal neg. affect + autonomic events	257.98	279.58	0.05	0.95	0.00	0.55	32.64%
3. Severity of SI, Contemporaneous Analyses							
High arousal neg. affect + autonomic events	9506.82	9541.21	59.13	0.80	0.02	1.87	97.00%
Low arousal neg. affect + autonomic events	9502.15	9542.27	34.69	0.81	0.02	1.86	93.02%
Low arousal neg. affective states	9490.10	9524.47	0.23	0.81	0.02	1.87	76.05%
High arousal neg. affective states	9493.14	9521.79	1.00	0.80	0.02	1.87	74.08%
Autonomic events only	9679.81	9702.73	0.00	0.79	0.00	1.96	0.00%
4. Severity of SI, Prospective Analyses							
High arousal neg. affective states	9520.71	9549.37	1.00	0.80	0.02	1.88	86.05%
High arousal neg. affect + autonomic events	9509.93	9544.32	0.07	0.80	0.02	1.87	61.00%
Low arousal neg. affect + autonomic events	9504.52	9544.65	0.01	0.81	0.02	1.86	59.40%
Low arousal neg. affective states	9517.92	9552.31	0.05	0.80	0.02	1.87	56.66%
Autonomic events only	9715.51	9738.44	0.52	0.78	0.00	1.96	22.99%

Bayes factor reference model was model with all self-report included.

*AIC* Akaike information criterion, *BIC* Bayesian information criterion, *RMSE* root mean squared error.

#### Severity of suicidal thinking

The third section of Table 2 shows contemporaneous (i.e., same hour) models and the fourth section of Table 2 shows prospective (i.e., next hour) models using severity of suicidal thinking as the outcome. With only one exception, adding autonomic events improved model fit. The only exception was in the prospective models, adding autonomic events worsened model fit for the model with high arousal negative affect (25.05% poorer performance). There were mixed findings regarding our hypothesis around greater relative improvements for low vs. high arousal negative affect. Contrary to our hypothesis, adding autonomic events to the contemporaneous models improved the model with high arousal (22.92% improvement) more than the model with low arousal (16.97% improvement). In line with our hypothesis (albeit weakly) adding autonomic events data only slightly improved the prospective model with low arousal negative affect, though only slightly (2.74% improvement). See sections 2 and 3 of Supplementary Table 2 for individual regression results.

## Discussion

The study yielded several key findings. First, variability in autonomic arousal (in the form of EDA, used to identify autonomic events) was associated with periods of suicidal thinking. Second, models with *only* physiological data, however, were generally weaker than models with *only* self-report data. This may be expected given the shared method of assessment (self-report EMA) for the affective states and suicidal thinking outcomes. Third, physiological data generally added to the ability of self-report data to predict the *severity* but not the *occurrence* of suicidal thinking. It is unclear why physiological data would improve prediction of severity but not occurrence. One possible explanation may be that suicidal thoughts come and go involuntarily throughout the day (for those who have such thoughts), but when people are distressed and in a high-arousal state, those thoughts become more severe (and possibly more persistent, although we did not test that in this study). Fourth, when predicting severity of suicidal thinking, physiological data generally improved model fit more for models with less conceptually overlapping self-report data (i.e., low arousal negative affect) than for overlapping self-report data (i.e., high arousal negative affect).

Taken together, our findings suggest that physiological data are most useful in detecting severity (but not presence) of suicidal thinking when paired with self-report data. However, there are some cases where physiological data may be useful in isolation. For example, because almost 75% of the autonomic events in this study occurred outside of a time when we had self-report data, wearable devices may be particularly useful to identify suicidal thinking that is not otherwise captured by self-report, which can be cumbersome to complete many times per day.

Although these findings are promising for our ability to improve the prediction of severity of suicidal thinking, several challenges remain. First, our conceptualization of autonomic events used a proprietary algorithm that has only been validated in one internal study by the device manufacturer. Thus, our study was testing the validity of the algorithm to detect autonomic events just as much as it was testing the construct of autonomic arousal, although findings appear positive for both. Second, increased autonomic arousal is not an exclusive indicator of psychological distress. Autonomic arousal is non-specific to the valence of emotion. Consequently, increased autonomic activity could also be an indication of emotion like surprise and excitement. Third, physical activity or heat also increase EDA in similar ways to the increases associated with autonomic arousal. To address both the second and third issue, work is needed to further refine the algorithm used to detect distress. Work by Wilhelm et al. [30] incorporates multiple data streams (e.g., accelerometer, EDA) to determine whether an increase is skin conductance is due to physiological arousal or a factor like excess movement. Finally, although we had a large number of observations per person, we had relatively small number of participants. Future studies should replicate these initial findings in samples with more participants.

In sum, the findings here show that autonomic arousal is a potentially useful data stream to aid in the prediction of severity of suicidal thinking. These data are less useful when predicting the presence of suicidal thinking. Being able to detect the severity of distress and suicidal thinking presents future directions for both prediction and prevention of suicidal thinking. Regarding prediction of suicidal thinking, passively detected autonomic arousal may be particularly relevant as an indicator of acute psychological distress or agitation, a key feature in theories of suicide such as Acute Suicidal Affective Disturbance [31]. Regarding prevention, passively detected autonomic arousal could enable us to deliver assistance in real-time when it is needed most. This may be a useful framework for knowing which intervention to deliver at which times, for example delivering a less cognitively-taxing intervention during times of higher distress. Such a framework may be particularly useful for individuals who are at risk for suicide but lack the insight to know when they were in distress (e.g., children who find wearable devices like the ones used in this study especially appealing [32]). Finally, wearable devices that detect autonomic events can likely be used in a variety of contexts beyond suicide (e.g., prevention of nightmares among those with Posttraumatic Stress Disorder [33]).

## Supplementary information

Supplemental Table
