# Supplementary material for: Can passive measurement of physiological distress help better predict suicidal thinking?
Source: Transl Psychiatry. 2021 Dec 2;11:611. doi: 10.1038/s41398-021-01730-y (PMC8640041; doi:10.1038/s41398-021-01730-y)
Supplement: Supplementary file 1 — Supplemental Table [file 41398_2021_1730_MOESM1_ESM.docx]

**Table S1. Results of the multi-level models for daily-level data**

| 1. Presence/Absence of SI, Contemporaneous Analyses | | | | | | | | | | | | | | | | | | | |
| --- | --- | --- | --- | --- | --- | --- | --- | --- | --- | --- | --- | --- | --- | --- | --- | --- | --- | --- | --- |
|  | **High arousal NA** | | | | | **Low arousal NA** | | | | **High arousal NA + Autonomic** | | | **Low arousal NA + Autonomic** | | | **Autonomic only** | | | |
| *Predictors* | *OR* | | *CI* | | *p* | *OR* | | *CI* | *p* | *OR* | *CI* | *p* | *OR* | *CI* | *p* | *OR* | *CI* | *p* |  |
| (Intercept) | 195.46 | | 0.04 – 1.08x10^6^ | | .230 | 2692.42 | | 2.73 – 2.66x10^6^ | **.025** | 153.83 | 0.04 – 5.43x10^5^ | .227 | 2439.89 | 2.22 – 2.68x10^6^ | **.029** | 40.44 | 0.65 – 2532.17 | .080 |  |
| Agitated | 1.45 | | 0.97 – 2.19 | | .072 |  | |  |  | 1.43 | 0.95 – 2.14 | .087 |  |  |  |  |  |  |  |
| Angry | 1.78 | | 1.01 – 3.11 | | **.045** |  | |  |  | 1.75 | 1.00 – 3.05 | .050 |  |  |  |  |  |  |  |
| Hopeless |  | |  | |  | 2.62 | | 1.60 – 4.28 | **<.001** |  |  |  | 2.57 | 1.57 – 4.23 | **<.001** |  |  |  |  |
| Fatigued |  | |  | |  | 0.64 | | 0.43 – 0.96 | **.031** |  |  |  | 0.65 | 0.43 – 0.97 | **.035** |  |  |  |  |
| Burdensome |  | |  | |  | 1.40 | | 0.94 – 2.09 | .098 |  |  |  | 1.39 | 0.93 – 2.07 | .104 |  |  |  |  |
| Autonomic |  | |  | |  |  | |  |  | 1.05 | 0.92 – 1.19 | .493 | 1.03 | 0.89 – 1.20 | .678 | 1.09 | 0.96 – 1.23 | .172 |  |
| 2. Presence/Absence of SI, Prospective Analyses | | | | | | | | | | | | | | | | | | | |
|  | | **High arousal NA** | | | | **Low arousal NA** | | | | **High arousal NA + Autonomic** | | | **Low arousal NA + Autonomic** | | | **Autonomic only** | | | |
| *Predictors* | | *OR* | | *CI* | *p* | *OR* | | *CI* | *p* | *OR* | *CI* | *p* | *OR* | *CI* | *p* | *OR* | *CI* | *p* |  |
| (Intercept) | | 4145.15 | | 29.60 – 5.80x10^5^ | **.001** | 2.02x10^3^ | | 2.01x10^3^ – 2.01x10^4^ | **<.001** | 3169.94 | 19.66 – 5.11x10^5^ | **.002** | 15918.53 | 160.89 – 1.57x10^5^ | **<.001** | 562.85 | 0.13 – 2.53x10^6^ | .140 |  |
| Agitated | | 1.36 | | 0.83 – 2.22 | .222 |  | |  |  | 1.29 | 0.79 – 2.12 | .311 |  |  |  |  |  |  |  |
| Angry | | 2.45 | | 1.20 – 4.99 | **.014** |  | |  |  | 2.42 | 1.18 – 4.99 | **.016** |  |  |  |  |  |  |  |
| Hopeless | |  | |  |  | 4.07 | | 4.06 – 4.07 | **<.001** |  |  |  | 3.92 | 1.93 – 7.98 | **<.001** |  |  |  |  |
| Fatigued | |  | |  |  | 0.58 | | 0.58 – 0.58 | **<.001** |  |  |  | 0.59 | 0.36 – 0.95 | **.030** |  |  |  |  |
| Burdensome | |  | |  |  | 1.40 | | 1.39 – 1.40 | **<.001** |  |  |  | 1.37 | 0.84 – 2.23 | .203 |  |  |  |  |
| Autonomic | |  | |  |  |  | |  |  | 1.10 | 0.94 – 1.29 | .255 | 1.10 | 0.91 – 1.33 | .346 | 1.14 | 0.98 – 1.32 | .081 |  |
| 3. Severity of SI, Contemporaneous Analyses | | | | | | | | | | | | | | | | | | | |
|  | | **High arousal NA** | | | | | **Low arousal NA** | | | **High arousal NA + Autonomic** | | | **Low arousal NA + Autonomic** | | | **Autonomic only** | | | |
| *Predictors* | | *b* | | *CI* | *p* | | *b* | *CI* | *p* | *b* | *CI* | *p* | *b* | *CI* | *p* | *b* | *CI* | *p* |  |
| (Intercept) | | 3.90 | | 2.48 – 5.31 | **<.001** | | 3.90 | 2.49 – 5.31 | **<.001** | 3.70 | 2.26 – 5.14 | **<.001** | 3.65 | 2.20 – 5.10 | **<.001** | 3.60 | 2.16 – 5.05 | **<.001** |  |
| Agitated | | 0.43 | | 0.27 – 0.58 | **<.001** | |  |  |  | 0.42 | 0.26 – 0.57 | **<.001** |  |  |  |  |  |  |  |
| Angry | | 0.52 | | 0.37 – 0.67 | **<.001** | |  |  |  | 0.50 | 0.35 – 0.65 | **<.001** |  |  |  |  |  |  |  |
| Hopeless | |  | |  |  | | 0.50 | 0.36 – 0.63 | **<.001** |  |  |  | 0.47 | 0.33 – 0.60 | **<.001** |  |  |  |  |
| Fatigued | |  | |  |  | | 0.07 | -0.06 – 0.20 | .290 |  |  |  | 0.07 | -0.05 – 0.20 | .259 |  |  |  |  |
| Burdensome | |  | |  |  | | 0.28 | 0.15 – 0.41 | **<.001** |  |  |  | 0.30 | 0.17 – 0.43 | **<.001** |  |  |  |  |
| Autonomic | |  | |  |  | |  |  |  | 0.08 | 0.03 – 0.12 | **.001** | 0.09 | 0.05 – 0.14 | **<.001** | 0.11 | 0.06 – 0.16 | **<.001** |  |
| 4. Severity of SI, Prospective Analyses | | | | | | | | | | | | | | | | | | | |
|  | | **High arousal NA** | | | | | **Low arousal NA** | | | **High arousal NA + Autonomic** | | | **Low arousal NA + Autonomic** | | | **Autonomic only** | | | |
| *Predictors* | | *b* | | *CI* | *p* | | *b* | *CI* | *p* | *b* | *CI* | *p* | *b* | *CI* | *p* | *b* | *CI* | *p* |  |
| (Intercept) | | 3.73 | | 2.38 – 5.08 | **<.001** | | 3.73 | 2.38 – 5.07 | **<.001** | 3.52 | 2.13 – 4.90 | **<.001** | 3.47 | 2.09 – 4.86 | **<.001** | 3.39 | 2.03 – 4.76 | **<.001** |  |
| Agitated | | 0.31 | | 0.13 – 0.49 | **.001** | |  |  |  | 0.30 | 0.12 – 0.48 | **.001** |  |  |  |  |  |  |  |
| Angry | | 0.44 | | 0.27 – 0.61 | **<.001** | |  |  |  | 0.42 | 0.25 – 0.59 | **<.001** |  |  |  |  |  |  |  |
| Hopeless | |  | |  |  | | 0.40 | 0.26 – 0.54 | **<.001** |  |  |  | 0.37 | 0.23 – 0.51 | **<.001** |  |  |  |  |
| Fatigued | |  | |  |  | | 0.05 | -0.09 – 0.18 | .511 |  |  |  | 0.04 | -0.09 – 0.18 | .532 |  |  |  |  |
| Burdensome | |  | |  |  | | 0.21 | 0.08 – 0.35 | **.002** |  |  |  | 0.24 | 0.10 – 0.37 | **.001** |  |  |  |  |
| Autonomic | |  | |  |  | |  |  |  | 0.08 | 0.03 – 0.12 | **.001** | 0.09 | 0.05 – 0.14 | **<.001** | 0.10 | 0.05 – 0.15 | **<.001** |  |

**Table S2. Results of the multi-level models for hourly-level data**

| 1. Presence/Absence of SI, Contemporaneous Analyses | | | | | | | | | | | | | | | | | | | |
| --- | --- | --- | --- | --- | --- | --- | --- | --- | --- | --- | --- | --- | --- | --- | --- | --- | --- | --- | --- |
|  | **High arousal NA** | | | | | **Low arousal NA** | | | | **High arousal NA + Autonomic** | | | **Low arousal NA + Autonomic** | | | **Autonomic only** | | | |
| *Predictors* | *OR* | | *CI* | | *p* | *OR* | | *CI* | *p* | *OR* | *CI* | *p* | *OR* | *CI* | *p* | *OR* | *CI* | *p* |  |
| (Intercept) | 21.23 | | 0.84 – 537.62 | | .064 | 28.29 | | 0.71 – 1120.42 | .075 | 20.24 | 0.80 – 511.35 | .068 | 27.09 | 0.69 – 1057.82 | .078 | 17.35 | 0.83 – 364.38 | .066 |  |
| Agitated | 1.52 | | 1.34 – 1.74 | | **<.001** |  | |  |  | 1.53 | 1.34 – 1.75 | **<.001** |  |  |  |  |  |  |  |
| Angry | 1.09 | | 0.93 – 1.27 | | .283 |  | |  |  | 1.08 | 0.92 – 1.27 | .341 |  |  |  |  |  |  |  |
| Hopeless |  | |  | |  | 1.78 | | 1.52 – 2.08 | **<.001** |  |  |  | 1.77 | 1.51 – 2.07 | **<.001** |  |  |  |  |
| Fatigued |  | |  | |  | 0.9 | | 0.79 – 1.02 | .110 |  |  |  | 0.9 | 0.79 – 1.03 | .121 |  |  |  |  |
| Burdensome |  | |  | |  | 1.38 | | 1.21 – 1.58 | **<.001** |  |  |  | 1.39 | 1.22 – 1.58 | **<.001** |  |  |  |  |
| Autonomic |  | |  | |  |  | |  |  | 1.35 | 0.97 – 1.87 | .078 | 1.25 | 0.88 – 1.78 | .221 | 1.34 | 0.99 – 1.83 | .058 |  |
| 2. Presence/Absence of SI, Prospective Analyses | | | | | | | | | | | | | | | | | | | |
|  | | **High arousal NA** | | | | **Low arousal NA** | | | | **High arousal NA + Autonomic** | | | **Low arousal NA + Autonomic** | | | **Autonomic only** | | | |
| *Predictors* | | *OR* | | *CI* | *p* | *OR* | | *CI* | *p* | *OR* | *CI* | *p* | *OR* | *CI* | *p* | *OR* | *CI* | *p* |  |
| (Intercept) | | 30.43 | | 0.02 – 57786.87 | .375 | 34.7 | | 0.01 – 103920.09 | .385 | 29.36 | 0.02 – 51542.42 | .375 | 32.83 | 0.01 – 80588.13 | .381 | 23.47 | 0.05 – 11393.81 | .317 |  |
| Agitated | | 1.31 | | 0.97 – 1.76 | .081 |  | |  |  | 1.29 | 0.95 – 1.75 | .097 |  |  |  |  |  |  |  |
| Angry | | 0.86 | | 0.61 – 1.21 | .385 |  | |  |  | 0.86 | 0.61 – 1.21 | .384 |  |  |  |  |  |  |  |
| Hopeless | |  | |  |  | 1.32 | | 0.93 – 1.87 | .117 |  |  |  | 1.3 | 0.91 – 1.85 | .151 |  |  |  |  |
| Fatigued | |  | |  |  | 0.84 | | 0.66 – 1.07 | .152 |  |  |  | 0.84 | 0.66 – 1.07 | .164 |  |  |  |  |
| Burdensome | |  | |  |  | 1.11 | | 0.87 – 1.42 | .384 |  |  |  | 1.13 | 0.88 – 1.44 | .339 |  |  |  |  |
| Autonomic | |  | |  |  |  | |  |  | 1.13 | 0.59 – 2.18 | .713 | 1.23 | 0.60 – 2.50 | .577 | 1.23 | 0.64 – 2.34 | .532 |  |
| 3. Severity of SI, Contemporaneous Analyses | | | | | | | | | | | | | | | | | | | |
|  | | **High arousal NA** | | | | | **Low arousal NA** | | | **High arousal NA + Autonomic** | | | **Low arousal NA + Autonomic** | | | **Autonomic only** | | | |
| *Predictors* | | *b* | | *CI* | *p* | | *b* | *CI* | *p* | *b* | *CI* | *p* | *b* | *CI* | *p* | *b* | *CI* | *p* |  |
| (Intercept) | | 3.82 | | 2.35 – 5.30 | **<.001** | | 3.82 | 2.35 – 5.30 | **<.001** | 3.77 | 2.29 – 5.24 | **<.001** | 3.76 | 2.29 – 5.24 | **<.001** | 3.76 | 2.28 – 5.23 | **<.001** |  |
| Agitated | | 0.31 | | 0.24 – 0.37 | **<.001** | |  |  |  | 0.31 | 0.25 – 0.38 | **<.001** |  |  |  |  |  |  |  |
| Angry | | 0.25 | | 0.18 – 0.32 | **<.001** | |  |  |  | 0.24 | 0.17 – 0.31 | **<.001** |  |  |  |  |  |  |  |
| Hopeless | |  | |  |  | | 0.31 | 0.26 – 0.37 | **<.001** |  |  |  | 0.31 | 0.25 – 0.37 | **<.001** |  |  |  |  |
| Fatigued | |  | |  |  | | 0.06 | 0.01 – 0.12 | **.020** |  |  |  | 0.06 | 0.01 – 0.12 | **.017** |  |  |  |  |
| Burdensome | |  | |  |  | | 0.13 | 0.07 – 0.19 | **<.001** |  |  |  | 0.14 | 0.08 – 0.20 | **<.001** |  |  |  |  |
| Autonomic | |  | |  |  | |  |  |  | 0.34 | 0.19 – 0.50 | **<.001** | 0.36 | 0.21 – 0.51 | **<.001** | 0.38 | 0.22 – 0.54 | **<.001** |  |
| 4. Severity of SI, Prospective Analyses | | | | | | | | | | | | | | | | | | | |
|  | | **High arousal NA** | | | | | **Low arousal NA** | | | **High arousal NA + Autonomic** | | | **Low arousal NA + Autonomic** | | | **Autonomic only** | | | |
| *Predictors* | | *b* | | *CI* | *p* | | *b* | *CI* | *p* | *b* | *CI* | *p* | *b* | *CI* | *p* | *b* | *CI* | *p* |  |
| (Intercept) | | 4.24 | | 2.38 – 6.09 | **<.001** | | 4.23 | 2.41 – 6.06 | **<.001** | 4.18 | 2.33 – 6.03 | **<.001** | 4.17 | 2.35 – 5.99 | **<.001** | 4.12 | 2.32 – 5.92 | **<.001** |  |
| Agitated | | 0.21 | | 0.06 – 0.35 | **.005** | |  |  |  | 0.2 | 0.06 – 0.35 | **.006** |  |  |  |  |  |  |  |
| Angry | | 0.1 | | -0.05 – 0.25 | .194 | |  |  |  | 0.09 | -0.06 – 0.24 | .251 |  |  |  |  |  |  |  |
| Hopeless | |  | |  |  | | 0.22 | 0.10 – 0.35 | **<.001** |  |  |  | 0.22 | 0.09 – 0.34 | **.001** |  |  |  |  |
| Fatigued | |  | |  |  | | -0.09 | -0.20 – 0.02 | .113 |  |  |  | -0.09 | -0.20 – 0.02 | .107 |  |  |  |  |
| Burdensome | |  | |  |  | | 0.09 | -0.03 – 0.21 | .128 |  |  |  | 0.11 | -0.01 – 0.23 | .082 |  |  |  |  |
| Autonomic | |  | |  |  | |  |  |  | 0.28 | -0.06 – 0.62 | .102 | 0.36 | 0.03 – 0.70 | **.032** | 0.34 | 0.01 – 0.68 | **.046** |  |
